# Comparing Indigenous and public health infant feeding recommendations in Peru: opportunities for optimizing intercultural health policies

**DOI:** 10.1186/s13002-018-0271-2

**Published:** 2018-11-20

**Authors:** Madalena Monteban, Valeria Yucra Velasquez, Benedicta Yucra Velasquez

**Affiliations:** 1 Department of Anthropology, University of Georgia, Athens, USA; 2 Scientific and Technical Research Council (CONICET), Buenos Aires, Argentina; 3 Asociación ANDES, Street Ciro Alegria H-13, Urb. Santa Monica–Wanchaq, Postal Nº 567, Cusco, Peru; 4 4600 S.S. de Jujuy, Argentina

**Keywords:** Andes, Infant feeding, Breastfeeding, Ethnomedicine, Intercultural health, Public health

## Abstract

**Background:**

The problem of childhood undernutrition in low-income countries persists despite long-standing efforts by local governmental and international development agencies. In order to address this problem, the Peruvian Ministry of Health has focused on improving access to primary healthcare and providing maternal and child health monitoring and education. Current maternal-child health policies in Peru introduce recommendations that are in some respect distinct from those of Indigenous highland communities. This paper analyses the similarities and differences between public health and mothers’ infant feeding recommendations. Furthermore, it analyses persistence and change in those recommendations among women who were mothers before and after the introduction of current public health policies.

**Methods:**

Semi-structured interviews were conducted with 18 older mothers, 15 currently breastfeeding mothers, and 15 public health staff in highland rural communities of Peru. During data analysis, thematic codes and text passages were used in an iterative analytic process to document emerging themes.

**Results:**

The results highlight the existence of a traditional corpus of beliefs surrounding infant feeding and care that is consistent with Andean ethnomedical beliefs. This is illustrated by mother’s accounts referring to the importance of maintaining a dietary balance of fluids and semi-fluids and of maintaining harmony with the elements in the natural environment. Mothers also incorporate aspects of public health recommendations that they find useful including initiating breastfeeding immediately after birth and exclusive breastfeeding up until 6 months. There are also tensions between the two systems including differences in the conceptualization of breastfeeding and infant food, the imposition of public health care services by coercive means, and negative stereotyping of rural Andean diets and mothers.

**Conclusions:**

Identifying similarities and differences between distinct systems may provide useful input for effective intercultural health policies. Sources of tension should be carefully assessed with the aim of improving public health policies. Such efforts should apply a process of cultural humility engaging health care professionals in exchange and conversations with patients and communities acknowledging the assumptions and beliefs that are embedded in their own understanding. This process should also recognize and value the knowledge and practices of Andean mothers and their role as primary caretakers.

**Electronic supplementary material:**

The online version of this article (10.1186/s13002-018-0271-2) contains supplementary material, which is available to authorized users.

## Background

Researchers in the Andes have long recognized the co-existence of Andean and biomedical health systems, where the use of home remedies and practices exist alongside traditional (e.g., shamans, bonesetters, midwives) and biomedical practitioners [1–6]. It is widely understood that exchange occurs between these health systems [3, 6–8]. Some authors stress the integration and syncretism of medical knowledge practices in the Andes [3, 9]. Other researchers affirm that traditional and biomedical systems in the Andes complement each other, as exemplified by multiple uses of both types of medicine [6, 7]. Past research has identified tensions between the two systems stemming from the dominant role of biomedicine, as sponsored by the state [10–16]. Current maternal-child health policies in Peru incorporate an intercultural framework, in an effort to respect traditional practices like vertical birth [7, 8] and the use of natural galactagogues^1^ [9]. However, public health policies are based on a system of knowledge that is meaningfully distinct from those of the Indigenous highland communities. The knowledge that mothers possess and employ is an important, yet largely overlooked, element in the analysis of traditional medical and nutritional systems [2, 17]. In the Andes, traditional maternal knowledge relating to infant feeding and health includes knowledge of specific plants, animals, minerals, and broader patterns of categorization that are structured by the particular worldviews within which mothers are embedded [18–20]. This research expands on the neglected topic of Andean maternal knowledge and infant care with the aim of identifying potential sources of tension and identifying venues for the complementation of distinct systems.

### Maternal and child nutrition in Peru

Child undernutrition is a major public health concern in Peru, where there are stark rural and urban differences. Forty-four percent of children in rural areas are stunted as compared to 16% in urban areas [21]. In order to address this problem, the Peruvian Ministry of Health has focused on improving access to primary healthcare and providing maternal and child health monitoring and education. In 2004, health clinics in the district of Cusco adopted the Peruvian Ministry of Health’s resolution *Lineamientos de Nutricion Materna* (Norms for Maternal Nutrition) to promote maternal and infant health [22]. This initiative mandates mothers receive pre- and postnatal monitoring and participate in nutrition workshops. Prior to the changes in policy and regulations, mothers either gave birth and accessed general maternal and child health services at the nearest public health post or managed birth and infant feeding at home with the assistance of family and local midwives.

Previous research in the Andes shows that the breastfeeding and nutritional practices of Andean mothers are guided by culture-specific beliefs. Researchers have identified how local conceptions of the body underlie specific infant feeding behaviors, emphasizing that mothers’ concepts of infant’s physical growth and development shape and guide infant feeding practices [23]. For example, mothers in Lima were more likely to breastfeed infants who suffered from linear growth stunting, diarrheal disease, and low intake of supplementary foods for a longer period of time than infants who did not suffer from these conditions [24]. Furthermore, in highland towns of Peru and Bolivia, mothers believe that their rage or sorrow is transmitted to their infants through breast milk [17, 25]. As a result, mothers under distress may temporarily alter their breastfeeding practices or stop breastfeeding altogether to avoid putting infants at risk [17]. Traditional practices also include the use of medicinal and nutritional properties of plants and animal that give the mother strength to recuperate from childbirth and are good to eat during breastfeeding [20, 26]. For example, plants, animals, and minerals have been identified throughout the Andean region that are incorporated into the diets of women during lactation, as they are believed to increase the production of breast milk [18, 20].

The introduction of maternal-child health policies, with services centered in public health clinics in Peru, offers an opportunity to examine the interplay between biomedical and Andean maternal-child health practices. This paper analyses similarities and differences in breastfeeding and postpartum nutrition beliefs and practices of public health clinics’ staff and of the mothers in local communities. Furthermore, it analyses persistence and change in those beliefs and practices among women who were mothers before and after the introduction of current public health policies.

## Methods

### Study location

The research was conducted in Cuyo Grande and Chawaytire, two Quechua rural communities in the Cusco Region, Peru. The communities are located between 3500 and 4500 m above sea level and have populations of 900 and 500 people, respectively. Chawaytire and Cuyo Grande share features of cultural and social organization with many communities in the area. They have a long history of interaction with non-governmental organizations (NGOs), Indigenous rights movements, local and regional markets, and tourism. Residents are subsistence farmers who grow potatoes (*Solanum tuberosum*), corn (*Zea mays*) and other Andean tubers and grains, and herd livestock. Residents supplement livelihoods with activities relating to crafts and seasonal migration for jobs. A public health clinic provides primary health care services in each community. Since 2002, pregnant women and children up to the age of five and living in conditions of poverty or extreme poverty receive health insurance coverage under the *Seguro Integral de Salud* (Comprehensive Health Insurance) program of the Peruvian Ministry of Health.

One of the pillars of the current strategy is to promote mother’s adherence to the WHO recommendations regarding breastfeeding to ensure adequate nutrition of children from birth to the first years of life. The recommendations of the WHO [27] include (1) a balanced diet of proteins, energy, and micronutrients necessary to support breastfeeding and infant growth, (2) exclusive breastfeeding for the first 6 months of life, (3) introduction of supplementary foods at 6 months, and (4) continued breastfeeding for 2 years or more. These practices are supported because they are associated with best maternal and child health outcomes [28, 29]. In addition, during workshops and check-ups, mothers receive information regarding perinatal diets including the introduction of solid foods. Recommendations include serving quantities, frequencies, and texture according to age. Public health staff recommend that animal origin foods such as meat, eggs, and milk are necessary for growth and avoiding illness. Diets should include foods rich in iron (such as liver and red meat), fruits and vegetables, and legumes every day.

Mothers who do not bring their children to regular growth and health controls may be denied access to health services in the future including the provision of monthly food rations. Mothers who do not give birth at a biomedical health clinic may be fined and some have reported problems obtaining a birth certificate for such births.

### Data collection

Data collection proceeded from December 2011 to December 2013 and consisted of conducting semi-structured interviews and observations. Semi-structured interviews were conducted among 18 mothers of over 45 years of age who had their children before the introduction of current maternal and child health policies and were identified by community members as knowledgeable about breastfeeding and child rearing. Another set of semi-structured interviews was conducted among 15 younger mothers who were currently breastfeeding and represented diverse characteristics regarding educational level, number of children, household composition, household income, and religion. Purposeful sampling was used to recruit participants. The interviews explored the mother’s breastfeeding perceptions and experiences with breastfeeding. We asked mothers to recall their experiences with breastfeeding and infant nutrition and discuss how they related to more general beliefs and behaviors (Additional file 1). Questions included the following: When does breastfeeding start? How often are infants breastfed throughout the day? What makes breastfeeding more or less difficult for the mother? Is breastfeeding exclusive? When are supplementary foods introduced? When does breastfeeding stop? What prompts a change in diet or breastfeeding? A third set of semi-structured interviews was conducted with an exhaustive sample of 15 public health staff at the local health establishments. The interviews explored breastfeeding recommendations and public health staff’s experiences regarding breastfeeding in the research communities. Questions for the public health staff included the following: What are the recommendations related to breastfeeding promoted by this establishment? When should breastfeeding be initiated? Why? For how long should breastfeeding continue? Why? Observations were conducted in the participants’ homes on four occasions of 12 h each, spanning from the child’s birth until reaching 1 year of age. The field notes contained information regarding the dynamics of child feeding and food consumption in the family. Observations were also conducted sporadically in the two public health clinics with the aim of learning about the interplay of public health service providers with the local clients.

The majority of participant mothers held some degree of Spanish language skills; however, Quechua was their primary and preferred language. A native field research assistant from Cuyo Grande who was bilingual in Quechua and Spanish collaborated in data collection. Interviews with public health staff were conducted in Spanish—their primary and preferred language. The semi-structured interviews were audio recorded and took between 20 and 60 min. A second research assistant transcribed and translated the interviews from Quechua to Spanish. Notes were taken during interviews and used to cross-check transcriptions to assure completeness and accuracy. In addition, a Quechua language scholar from the city of Cusco checked the accuracy of the translations.

The Institutional Review Board of the University of Georgia provided ethical approval to conduct this study and participants provided their informed consent. In addition, elected officials from the participating communities provided permission to conduct the research.

### Data analysis

The analysis of interviews followed an inductive reasoning using the narratives to build interpretations and meanings [30]. Organizing concepts and categories were identified with an open codification scheme [31]. This process implied assigning an open code (i.e., in-vivo code) for each relevant section of text in the transcripts to authentically represent the participants’ own words and capture emerging concepts. Example open codes included the following: colostrum for strength, colostrum for quick development, [human] milk for wounds, [human] milk for *costado,*^2^ [human] milk for lung. The text coding was developed with ATLAS.ti (6.2) [32]. Thematic codes and relevant text passages were used in an iterative analytic process to document emerging themes. Each category was then analyzed in detail, cross-checking coding strategies and interpretation of data between two independent analysts. Content disagreements were discussed and the emerging insights provided for refining coding frames [33]. Emerging themes were diverse and encompassed narratives about the transmission of knowledge regarding description and interpretation of the forms of use, involvement of household members, and perceptions about breastfeeding and postpartum maternal and child diets effects. The consistency of results was cross-checked with published data on Andean ethnomedical concepts [19, 34–36] and through discussion with community members and research assistants [37].

The household observation data was used to cross check the results from the semi-structured interviews. Field notes from observations in health clinics were analyzed to examine the involvement of clinics’ personnel in promoting WHO breastfeeding recommendations.

## Results

Below, we present six key themes of similarities and differences between the views of mothers 45 years and older and public health staff. Table 1 includes brief summaries of these key themes. The views and practices of currently breastfeeding mothers are incorporated in a final section where we present convergences and tensions between the Andean and public health infant health systems. Participant names are pseudonyms.

**Table 1:** Differences and similarities between the infant feeding recommendations of public health staff and Andean mothers

	Public health staff	Andean mothers (45 years and older)
Appraisal of breast milk properties	-Immunological defenses help protect infants from illnesses -Balance between macro and micronutrients up until 6 months of age	-Provides strength and helps infant rapidly develop -Breast milk is a “cold” medicine used to cure “hot” illnesses such as *costado*, backache, eye infections, and “hot” fevers
Breastfeeding initiation	-Initiation as soon as possible after birth to establish proper breastfeeding and a strong bond between mother and infant -Beneficial for mother (contraction of the uterus) and child (psychological and development health)	-Follow infant cues to initiate breastfeeding -Infant is full afterbirth and needs rest
Perinatal diet, breastfeeding, and nutrition	-Perinatal diets need to be high in protein and micronutrients (iron and vitamin A)	-Balance of hot, cold, wet, and dry foods provide strength, return mothers to health, and help produce nourishing breast milk
Introduction of solid foods	-Receiving solid foods prior to 6 months of age = higher incidence of respiratory illness and diarrhea -Illnesses suppress appetites and disrupt normal growth patterns	-Foods are introduced in response to infant cues -Infant is hungry or infant is “looking” at the foods -Experience of cravings and hunger increase children’s susceptibility to illness
Breastfeeding cessation	-Breast milk after 2 years does not have the same consistency	-Follow child cues to determine breastfeeding cessation -However, if breastfeeding continues beyond 1 year and 6 months, the child will be “loco” (crazy) “big-hearted” (feisty), “mañoso” (spoiled)
Exposure to environmental factors	-Environmental factors such as cold and wind increase children’s susceptibility to illnesses such as upper respiratory infections and the flu	-Elements in the landscape (heat, cold, bad-wind, *pujyo*, and *paq’o*) enter the breast and “spoil” breast milk and pass on illness to the infant

### Appraisal of breast milk properties

Results from interviews with public health staff and older mothers showed that both highly valued nutritional and medicinal properties of milk. Mothers referred, for example, to colostrum as an important source of nourishment for infants, providing strength. This belief is exemplified by the following quotation:

“We give colostrum, all, the baby breastfeeds everything, so that it is strong. It is the same in cows, we make the calves drink its mother’s colostrum so that it’s strong, and also its development is quick, and it will be strong at work.” (Lorena, mother, 63 years old)

In addition, for mothers, breast milk is more than infant food. It is identified as a “cold” medicine that is used to cure “hot” illnesses such as *costado*, cough, backache, eye infections, and “hot” fevers. The following are representative statements:

“Milk, human milk, for wounds…Quickly with a person’s milk it [a wound] heals…Also for lung, it [human milk] is also good right! When the lung is breathing with difficulty, then you have [the person whose is sick] drink the boy’s milk, you rub, so then quickly the person reacts. … also human milk is good for *costado*. You drink it inwardly, and you rub [on back], then, quickly it calms [eases symptoms].” (Ana, mother, 60 years old)

“When the baby’s eye is red you put a little bit of milk, it returns to normal, it is no longer red. When [the baby] has fever I also rub him with my milk. [Human] milk is fresco [cold], the milk takes away [the baby’s] heat.” (Olivia, mother, 55 years old)

The classification and use of human breast milk as a “cold” medicine to cure “hot” illnesses is consistent with Andean medicine’s humoral beliefs. As reported by several Andean scholars, illnesses and medicines are classified by their “hot” or “cold” humoral elements. Illnesses that are classified as hot are normally combated with herbal remedies that are “cold” and vice versa [19, 38, 39].

Public health staff discussed the nutritional properties of breast milk such as the proper balance between macro and micronutrients to meet nutritional and energy needs of infants. The immunological defenses that colostrum provides for infants were also emphasized. According to an obstetric nurse, “colostrum is like a first vaccination, the only thing that is needed for [the infant’s] health and good growth and development, to not have problems.” (Claudia, public health staff, 37 years old).

Other benefits ascribed to breastfeeding by public health staff included speeding up the contraction of the uterus for the mother after birth, strengthening the bond between mother and child, as well as general psychological and development health benefits for the infant. A typical way of statement follows:

“[breastfeeding] immediately after birth helps with the contraction of the uterus and that will prevent hemorrhaging” [and] “for the baby because of the bond, the affective relationship between mother and child.” (Carolina, public health staff, 43 years old)

### Breastfeeding initiation

Public health personnel highlighted the importance of initiating breastfeeding as soon as possible after birth. Ideally, if there are no complications, the newborn is given to the mother to initiate breastfeeding immediately after birth. The attending nurse obstetrician encourages the initiation of breastfeeding and gives the mother advice regarding breastfeeding techniques. Beginning breastfeeding as soon as possible after birth is seen as an important factor for establishing proper breastfeeding because mothers have access to health personnel readily available to guide them in the process. The following are representative statements:

“We teach breastfeeding techniques, how to hold the baby, posture, hold [the baby] with only one arm, make a C with your hand and grab the breast, make the baby play so that it opens its mouth and it grabs the entire dark part of the breast, posture, about the frequency, that it has to be 15 minutes on each breast at least. We teach that especially to the first time mothers…” (Elena, public health staff, 32 years old)

“as soon as the baby is born, in the cases where there are no complications for the baby we promote immediate contact, we take the baby to the mother’s breast 10 or 20 seconds after birth…” (Laura, public health staff, 28 years old)

However, older mothers who gave birth at home recalled following infant cues to initiate breastfeeding. After birth, the infant is viewed as being full from being nourished in the womb. Rather than being hungry, the infant is seen as needing rest. The following quote illustrates this point:

“[I began breastfeeding] after a day. They say the baby is born having drank lots of blood, because of that it is not hungry. After that [some hours when the baby becomes hungry again] then you give [breast milk] normal. When [the baby] is born, it sleeps deeply, does not wake, because of that people say… since [the baby] is born with a full belly, [the baby] eats blood inside [the mother’s] belly, [the baby] will not wake when [the baby] is just born, it is full of blood.” (Alicia, mother, 47 years old)

Reportedly, breastfeeding is initiated when the infant cries, cuing the mother to its hunger. For example, one mother explains why she immediately breastfed after birth: “I immediately give [breast milk] to my babies, because my babies were born crying” (Susana, mother, 69 years old). Initiation of breastfeeding usually occurs immediately after birth, a few hours, or even 1 or 2 days after birth according to infant cues.

### Perinatal diet, breastfeeding, and nutrition

Public health clinics’ staff and older mothers agreed on the importance of the postpartum diet. However, the overall perception of the clinics’ staff was that the dietary characteristics of the low-income population in highland communities are lacking in protein and energy and contribute to the high rates of child undernutrition. The staff considered that local diets presented deficiencies to meet the nutritional requirements of lactating mothers and to support infant growth and health, as it consists mainly of potatoes consumed in “watery” soups. Reported nutritional deficiencies included low protein, fat, and micronutrients (iron and vitamin A) content. Public health staff often mentioned that “the mother did not receive proper nutrition during pregnancy,” and that “the mother does not receive a proper diet during breastfeeding” (Linda, public health staff, 35 years old).

Nevertheless, older mothers considered that the diet is particularly important during the postpartum recovery period and during breastfeeding. Consumption of the appropriate foods reportedly returns a new mother to health and helps her to produce nourishing breast milk. Older mothers in the research sites emphasized the importance of maintaining strength (*fuerza*) both for the mother and child*.* Thus, the postpartum recovery period includes a regimen of prescribed and proscribed foods. Ideally, a sheep is slaughtered after birth to make a soup of lamb’s meat and innards cooked with *ch’uño* (freeze-dried potatoes*)*, *asnapas* (garnish such as oregano [*Origanum vulgare*], cilantro [*Coriandrum sativum*], and *wacatay* [*Tagetes minuta*]). This soup or any other food that may be given to the mother postpartum should be *chumo* (free of salt) as salt is said to fester the wounds caused during childbirth and impedes their healing. This is the ideal food for postpartum mothers, seen as best aiding the mother to regain her strength lost during childbirth, ward off illness, and produce breast milk. In agreement with the Andean concept of health, mothers perceive that a diet balanced in hot and cold and wet and dry properties helps protect against this weakness and promote strength [18, 26, 35, 39].

### Introduction of solid food for the breastfeeding infant

Following WHO recommendations, public health staff emphasized the importance of exclusive breastfeeding for the first 6 months of age of the child. Exclusive breastfeeding was defined as feeding no other foods or liquids to the baby except breast milk. Exclusive breastfeeding for the first 6 months is associated with optimal infant growth and development as breast milk contains all of the macro- and micronutrients that an infant needs with added immunological benefits [27]. Public health personnel described the perception that infants that are exclusively breastfed for the first 6 months of life get sick less. This is in concordance with biomedical evidence showing that infants that are exclusively breastfed for 6 months get sick less and follow an optimal growth trajectory [40–43]. They noted that infants who were fed foods or liquids before reaching 6 months are more likely to experience respiratory infections and diarrhea. These illnesses are in turn associated with fevers that likely suppress infant appetites and result in weight loss and generally disrupt normal growth patterns. They stated that introducing foods or liquids before the recommended 6 months of age was a factor influencing the high incidence of infant undernutrition in the highland communities. Public health staff expressed these concerns in the following manner:

“[before 6 months] the baby’s stomach is not ready to receive foods… they can cause damage.” (Marta, public health staff, 43 years old)

“[among babies that receive food before 6 months] sometimes [you see] diarrhea episodes, so we relate that, it can be because [the baby] already received other foods.” (Nora, public health staff, 29 years old)

“[diets have] animal products that are what make children grow, meat, milk, egg, fats, from 6 months of life, solid mashed-up foods, add a tea spoon of oil, that’s what helps growth.” (Gladys, public health staff, 37 years old)

For older mothers, complementary feeding (as with breastfeeding initiation) begins according to infant cues. The mother perceives that the infant is hungry or the infant is “looking” at the foods that the family is eating and seems interested in eating. The quotation below is a representative statement of this belief:

“From three months [the baby] already starts to have taste with a spoon or with your finger. [The baby] sees you eat; you have him [the baby] taste a little bit. From five months, [the baby] wants to eat and at six months [the baby] eats well, because the baby sees what you are eating and craves. That is why I have [the baby] taste. From five months [the baby] wants foods already and at six months [the baby] already eats well.” (Marina, mother, 60 years old)

Likewise, a balance of quantity of liquids and foods is considered by mothers important for health maintenance. Older mothers emphasized that proper diet and avoiding hunger are important aspects of health for infants. Too little food can deprive children of strength making them susceptible to illness. Too much food can also weaken children leaving them susceptible to illnesses. Correspondingly, children’s food cravings are indulged and their exposure to hunger is minimized as both are seen as increasing children’s susceptibility to illness.

Because infants have a tendency to express intense emotions, they are perceived to be more susceptible to illness. Therefore, mothers tend to indulge infants in order to avoid the expression of excessive negative emotions. This can be seen in the practice of on-demand breastfeeding, indulgence of child food preferences, and following child cues of the cessation of breastfeeding. In addition, older mothers expressed the belief that one should not breastfeed when angry because the anger will pass through breast milk to the child and cause an illness. An older mother expressed the following:

“I made them [my children] sick. I breastfed them [milk infected with] *calor interior* [internal heat]. [my baby’s] vomit was yellow. … my husband arrived drunk… so from getting mad at [her husband] … I breastfed my baby… and I made [my baby] sick with *colera* [anger that passed through with the breast milk].” (Ana, mother, 60 years old)

A common illness associated with infant feeding is *nilpo.* It is similar or equivalent to the illness *empacho* found throughout Latin America [44]. Interviews with older mothers suggest it is caused by either eating too much of one thing (too much “hot” or “cold” substances) or an unsatisfied food craving as indicated in the following statement:

“[my baby] got sick once [from *nilpo*] … I ate at a restaurant and I didn’t let [the baby] try a jello thinking that it would make her sick but the next day she was very sick….it must have been the [unsatisfied] craving [for jello].” (Nora, mother, 61 years old)

They listed symptoms of *nilpo* including stomach ache, diarrhea episodes one after the other mixed with some blood, internal gasses, and loss of appetite. Cures include eating toasted cheese with blackened spices, a drink made from *salvia* (sage) [*Salvia officinalis*] and other medicinal plants, and, in the case of an unsatisfied food craving, eating whatever was craved. The common practice of following infant cues to begin complementing breastfeeding with other foods is often explained as a means of avoiding an unsatisfied food craving and *nilpo*.

In addition, *nilpo* illness can be passed on to the infant through breast milk. Older mothers noted that whatever the mother eats passes to the breastfeeding infant through her milk. Breastfeeding mothers have to pay attention to the foods that they eat. It is necessary to balance hot and cold substances for too much of one type of substance can bring on *nilpo*.

As previously reported, experiencing excessive negative emotions, including anger and sadness were also perceived to increase a person’s susceptibility to illness.

“Sobbing and sighing are thought to allow *wayras* (winds) to enter through the eyes and mouth. Negative emotions cause the folk illness *mal de corazon* (heart sickness; also *mal de Corazon wayra),* […] and *colerina* (anger sickness). They also exacerbate other illnesses” [6 p.1011].

### Complementary breastfeeding

When asked whether breastfeeding should continue beyond the 2 years of age, public health personnel expressed their concern as follows:

“It is somewhat inconvenient [breast feeding beyond two years of age], because the child is going to start to become undernourished, because [the child] does not eat foods. [Breast milk after two years] does not have the same consistency, it becomes a complement that means that the child will at least have liquids… [Breast milk] continues to have vitamins, proteins but in small quantities, for what the child needs, and it protects against diseases.” (Doris, public health staff, 31 years old)

Older mothers considered child cues for the cessation of breastfeeding. However, the consensus for the ideal time to stop breastfeeding was when the child is a year and a half. Many older mothers stated that if the child is allowed to continue breastfeeding beyond a year and a half, he or she is likely to be “*loco”* (crazy), “big-hearted*”* (feisty), or “*mañoso”* (spoiled) negative attributes that will shape the child’s personality even as an adult. An older mother also expressed this view as follows:

“Until three years I gave [breast milk], only one of my children I gave [breast milk] until two years with two months. That one, my youngest child I also gave three years with three months. That is why my children now have a big heart, when they get mad they cannot stay quiet, they get angry easily, now when they are big. That is why it is not good to breastfeed until they are too big [three years], when someone bothers them they want to hit someone. I did not know that before that this would happen, now people tell me that you have to breastfeed one year and six months.” (Alicia, mother, 47 years old)

### Exposure to environmental factors

Public health staff were asked if they considered environmental factors such as hypoxia (deficiencies in the amount of oxygen delivered to the body tissues) related to problems with infant growth. However, public health staff attributed the high incidence of chronic undernutrition solely to deficiencies in local diets and suboptimal infant feeding practices. Upon probing regarding why infant growth faltering was not associated with living in a high altitude environment, one public health staff indicated knowing of stunting reversed in other rural low-income communities, for example, in China, where reversal occurred once diets incorporated more protein and energy dense foods.

On the other hand, the mothers were concerned about health problems caused by exposure to pathogenic forces in the landscape such as cold and heat as well as spirits. Mothers expressed the belief that when breast milk accumulates between suckling bouts (breastfeeding), it is likely to “spoil” as a result of exposure to elements in the landscape. Older mothers described the practice of expressing some breast milk before nursing as a preventive practice as follows:

“My mother said that one has to take out [breast milk] before breastfeeding. I would not take all of it [breast milk], only a little bit. In all of my children, I would always take out [breast milk], only a little bit.” (Berta, mother, 45 years old)

Illnesses mentioned by mothers that “spoil” breast milk include *paq’o*, wind, and *pujyo*. One mother explained the occurrence and treatment of *paq’o* and wind. Following is her description:

“I would always take some [breast milk] out because that is where illnesses come from like fever. When you sit in “hot” [exposed to the sun], from that [the child by way of breast milk] gets a fever with diarrhea, *paq’o*. [The baby then] has diarrhea like foam. I would cure [the baby] with *chili chili* [*Geranium filipes*], that is good for that. I gave all my children that *chili chili*. You boil [*chili chili*], you have the baby drink it also the mom drinks it. If it is from wind then [the baby] has a stomach ache, not diarrhea, [the baby] cries a lot. For that [wind] *mutuy* [*Senna birostris*] is good, heated in the hearth. You pass [the heated *mutuy*] over the [baby’s] body, or with semolina (coarse ground wheat), with that [the baby] heals.” (Lorena, mother, 63 years old)

These illnesses have been reported previously [6, 36, 45] however not in connection to breastfeeding and how they affect breast milk, i.e., anyone can fall ill from *paq’o* or *pujyo*; however, these illnesses affect the breastfeeding mother in a different manner for *paq’o* and *pujyo* can enter the breasts and affect the quality and production of breast milk. The course of treatment is therefore centered on curing the breast of the affected mother.

Furthermore, breast milk is considered a powerful substance, not to be disposed of lightly. When mothers express their milk following the belief that the accumulated milk has “spoiled,” they should be mindful of what they do with the milk that they extract. Even though the milk is extracted from the mother’s body, this does not mean that its connection to it is severed. If extracted breast milk is exposed to the hot rays of the sun, bad wind, or cold, it can cause the mother to fall ill. An older mother explained this practice as follows:

“When the baby does not breastfeed [when it is still a new-born], because it is still like out of it, I would take some [breast milk] out and I would throw it [the expressed milk] on the floor and I would bury it… I buried milk because when you throw it out like it is nothing the wind can pass and it [the breast] could hurt more and drip.” (Berta, mother, 45 years old)

### Convergence and tensions

While in some aspects traditional and biomedical health systems are complementary, there are sources of tensions between the two systems that may emerge from differences in the conceptualization of breastfeeding and infant food, from unmet expectations, and longstanding preconceptions.

In several aspects, the public health guidelines are being adopted by the mothers. Since 2007, mothers have been required by an ordinance of the Ministry of Health to birth at a public health establishment with the assistance of a physician. The majority (92%) of currently breastfeeding mothers stated that they initiated breastfeeding soon after birth as they are guided to do so by public health staff. Three currently breastfeeding mothers stated that they initiated breastfeeding 2 to 5 days after birth because of health complications (preterm birth, cesarean, and retained placenta). The traditional practice, however, indicated that mothers should follow the infant’s cues to initiate breastfeeding.

The majority of currently breastfeeding mothers (72% or 12 of 16) stated that they began introducing complementary foods at 6 months because “that is what the nurses tell us” (currently breastfeeding mother). The remaining 28% of mothers stated that they begin having their babies try foods and liquids other than breast milk prior to 6 months because “that is what my mother told me”, or “my baby started looking at [craving] foods before six months” (Dina, currently breastfeeding mother, 23 years old).

Some currently breastfeeding mothers discussed problems with infant growth, reflecting that mothers today are “taking better care of children because before they were skinny and they would not grow” (Sol, currently breastfeeding mother, 25 years old) or “the nurses told me that my youngest daughter was not reaching her height, her weight…. the nurses gave me a syrup (iron supplement) and *chispitas* (multi-vitamin powder) now she is fine” (Mari, currently breastfeeding mother, 23 years old). However, discussions regarding infant size are missing from older mothers’ narratives. One older mother states: “I am not interested that they [my children] be big but that they are healthy and strong” (Nilda, mother, 56 years old).

While in many aspects public health policies are being adopted by the mothers, potential sources of tensions emerged from the field work. These tensions include differences in the conceptualization of breastfeeding and infant food and the imposition of public health care services by coercive means. For example, mothers fear that if they do not bring their children to regular growth and health controls, they may be denied access to health services in the future including the provision of monthly food rations. One mother states “I bring my child to the health post because if you participate then they [public health] help you if you do not participate then they do not” (Selina, currently breastfeeding mother, 39 years old).

Another source of tension was associated with preconceptions of and the stereotyping of rural mothers. All public health staff were from nearby Andean urban centers where they received their higher education training in healthcare. Few public health staff (*n* = 5) indicated learning Quechua in the home growing up and the rest began to learn Quechua as a requirement to work in rural Quechua communities. Public health staff shared Andean cultural backgrounds and identities including beliefs, and practices such as the importance of consuming balanced diets during breastfeeding and the need to protect children from environmental factors present in high altitude environments such as cold and wind. However, the narratives of public health staff also reflected negative stereotyping of rural Andean diets and Andean mothers in comparison to urban Andean diets and mothers. For example, the staff is dubious about the medical and nutritional competency and the child-rearing priority of community mothers. One public health staff expressed this concern as follows:

“Mothers from the campo (rural communities) do not nourish [nourish their children] well, they give [them] too little nutritious food. While they are working during the day, from early in the morning they give [feed their children] only *ch’uño* [freeze dried potatoes]. People from the campo (rural communities) prioritize only the *chakra* (crops).” (Linda, public health staff, 35 years old)

The narratives of older mothers also expressed sources of tensions associated with differences in the conceptualization of breastfeeding and the timing of introduction of infant food as the following quote exemplifies:

“If my son wants to eat before six months I cannot wait for what they [public health staff] say. It is up to me, if my baby wants to eat something, I have to give food. Sometimes I do not pay attention to them [public health staff], maybe in other things, I can listen to them, but not in that [feeding before 6 months]. It is something natural for me, for example if I want to eat more of something and she [public health staff] forces me to eat less, to me that is not good.” (Ana, mother, 60 years old)

## Discussion

The results illustrate the existence of a traditional corpus of beliefs surrounding infant feeding and care that is consistent with Andean ethnomedical beliefs [19, 35, 46, 47]. This is illustrated by mother’s accounts referring to the importance of maintaining a dietary balance of fluids and semi-fluids and of maintaining harmony with the elements in the natural environment. Within this conceptual framework, the mothering expertise of women is recognized, and mothers in turn are keen on interpreting infants’ cues (i.e. weakness, hunger, cries) to avoid illness.

**Fig. 1:**
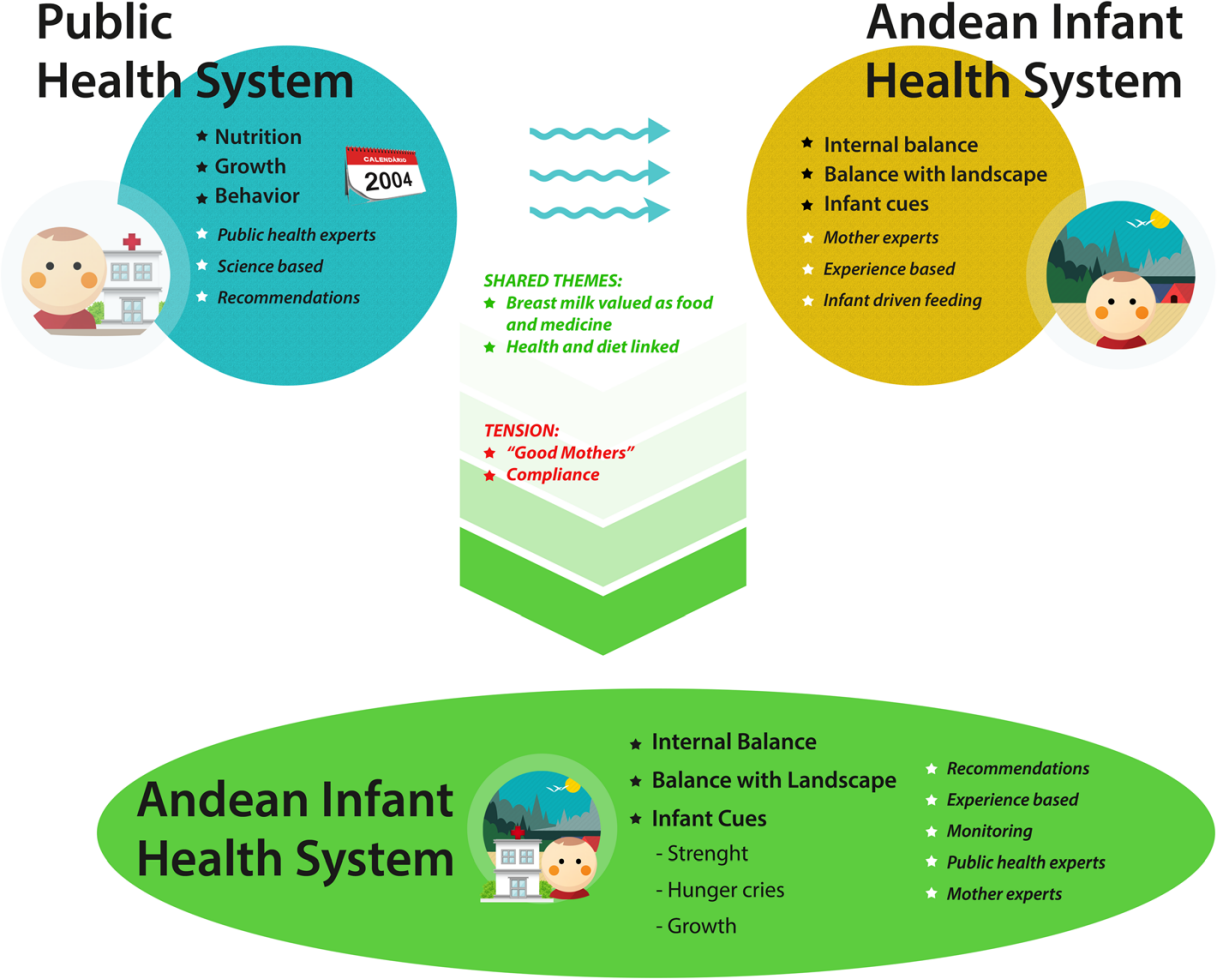
Resilience of the Andean infant health system. The public health system is represented in the blue circle and includes growth monitoring and dietary and behavioral recommendations introduced by maternal and child health initiatives of the Peruvian Ministry of Public Health in 2004. In this system, public health personnel are experts and policies and recommendations are based on scientific evidence. The Andean system, represented by the yellow circle, illustrates the existence of a traditional corpus of knowledge surrounding infant feeding and care consistent with Andean ethnomedical knowledge including the importance of dietary balance and maintaining harmony with elements in the natural environment. Within the Andean system, mothers are acknowledged as experience-based experts and respond to infant cues such as weakness, hunger, and cries. The two systems share themes including that breast milk is valued as both food and medicine and the knowledge that health and diet are intricately linked. However, there are also tensions between the two systems. The green shape at the bottom represents a resilient Andean infant health system. In the context of change, the Andean system has maintained key cultural elements while incorporating key public health recommendations. This figure was designed by the authors in response to the discussion presented in this manuscript

In the context of changes brought about by the implementation of maternal and child health policies, this research found that the Andean cultural system has absorbed disturbances by maintaining key cultural elements while incorporating recommendations of the public health system. The narratives of currently breastfeeding mothers point to the recognition of growth (length and weight) as an indicator of infant health. In addition, most mothers have incorporated key recommendations, like initiating breastfeeding immediately after birth, exclusive breastfeeding up until 6 months and continue breastfeeding and supplementary feeding, as well as attending infant growth and health monitoring at the local public health facilities (Fig. 1).

Mothers continue to pay attention to the influence of natural elements in the production of illnesses and to the incorporation of liquids in the diets to maintain proper balance. Interpreting and responding to infant cues, a practice that is not incorporated into WHO recommendations, is important for mothers in the research sites. Few studies have examined mothers’ response to infant cues and the relationship with breastfeeding. Newby and Davies [48] conducted a prospective study in Australia and concluded that health professionals can assist women to recognize cues of hunger and satiety in their infants and understand the dynamics and natural history of breastfeeding to prolong breastfeeding relationships. A study by Brown and Arnott [49] among parenting clinics attendees in the UK showed that an infant-led approach characterized by responding to and following infant cues was associated with longer breastfeeding duration. Following infant’s cues has also been reported to be associated with the prevention of early solid food introduction [50–52] and is not exclusive of Indigenous women’s mothering practices. Walsh, Kearney, and Dennis [53] reported that first-time mothers found that waiting until 6 months was challenging despite knowledge of the WHO recommendations and an initial desire to comply with this guideline. Mothers believed that complementary foods would assist the infants’ weight gain, sleeping patterns, and enjoyment at mealtime.

Knowledge systems, in general, are not static but change in response to dynamic processes using past and present experiences. In addition, by engaging with the public health system, mothers are ensuring access to multiple resources and expanding their knowledge base to buffer infant vulnerability. Norberg et al. [54] contend that diversity is the key to resilience. It is argued that knowledge systems are enhanced and not diminished by the meeting of two or more cultures, what Turner et al. [55] have termed the “cultural edge effect”. Exploring resilience of home-remedies among Bolivian and Peruvian migrants in England, Ceuterick and colleagues [56] site the presence of cultural diversity as tools in the maintenance and expansion of knowledge and use herbal-based home remedies.

The research found several points of convergence between the two systems. Breastfeeding is valued as the best source of food and medicine, infants and breastfeeding mothers are viewed as particularly vulnerable to disease, and there is an emphasis on the link between diets and health. The importance of perinatal diets for breastfeeding mothers and infants is in line with Gillespie [57] who found that food-based nutrition education was taken up by rural mothers in a central Andean community of Peru. The emphasis on balanced diets is informed by the Andean practice of consuming foods that provide strength and are balanced in hot, cold, wet, and dry properties. In addition, many of the foods consumed by mothers during breastfeeding are high in protein [18], consistent with the public health recommendation of consuming high protein and micronutrient-rich diets. These points of convergence are possible leverage points for intercultural health policies.

The research also found tensions between the two systems which are echoed in the findings of several researchers in varied Andean communities in Ecuador, Peru, and Bolivia [2, 6, 17, 57, 58]. Mathez-Stiefel et al. [33] in their examination of traditional and biomedicine in the Andes provide the following consideration:

“The encounter between various medical ideologies, far from being harmonious, is characterized by the tentative hegemony of biomedicine over indigenous medical traditions. This leads to changes in some health practices in Andean households but at the same time to a reflexive maintenance and revalorization of other aspects of indigenous medicine, in a process of cultural resistance and renewal.” (p.13)

Gillespie [57] reports tensions between public health staff and mothers in the central Peruvian Andes. These tensions include the stereotyping of Andean children as short and not intelligent in comparison to children raised in urban centers [57]. Similar to our own findings, these differences are attributed by public health staff to deficiencies in Andean diets. A “cultural” barrier is often assumed to be held by the patients, whereas in our findings and that of Gillespie [57], cultural beliefs surrounding perinatal diets are congruent and complementary with that of public health. Findings suggest that rather than mother’s beliefs surrounding perinatal diets, stereotyping of rural mothers and children is a potential cultural barrier in biomedical culture and practice.

The integration of biomedical and Indigenous health systems is fast becoming a more accepted approach throughout the world [59]. Intercultural health is understood as practices in health care that bridge Indigenous and Western medicine, where both are considered as complementary [60]. In 2016, the Ministry of Public Health introduced a new intercultural health legislation. Stipulations of this new legislation include the following: provide health as a human right for Indigenous and Andean peoples in all healthcare levels, institutionalize intercultural communication and dialog, recognize the importance and value of traditional medicine, and promote its articulation with conventional medicine [61]. The knowledge and practices of mothers is one component that should be considered as Peruvian health care incorporates an intercultural perspective.

Furthermore, capacity building, for the adequate implementation of an intercultural health strategy, has been identified as a salient component of health professionals’ education [62–68]. Research has shown that provider-patient communication is linked to patient satisfaction, adherence to medical instructions, and health outcomes [67]. Cultural humility is a capacity building tool that may assist in overcoming tensions in intercultural encounters. Cultural humility refers to an ongoing process that requires health care professionals to engage in exchange and conversations with patients, communities, colleagues and themselves [68]. It involves being trained to acknowledge the assumptions and beliefs that are embedded in their own understanding, rather than delving into the patient’s belief system [69]. This approach could assist in optimizing cross-cultural encounters between the perspective of public health professionals and of community mothers in Peru and perhaps other Andean healthcare settings.

## Conclusion

A traditional corpus of beliefs surrounding infant feeding and care is consistent with Andean ethnomedical beliefs. In this context of change, brought on by the institutionalization of perinatal care, the Andean system has maintained key cultural elements while incorporating key public health recommendations. Our findings reinforce the conception that knowledge systems are not static but change in response to dynamic temporal processes. Andean and biomedical recommendations endorse the value of breastfeeding as the best source of food and medicine and emphasize the benefits balanced perinatal diets have on nursing mothers and infants. Sources of tensions should be carefully assessed with the aim of improving public health policies. Such efforts should apply a self-reflexive process of cultural humility, wherein patients, health care practitioners, and communities alike are engaged in a manner that acknowledges the assumptions and beliefs embedded into their own understanding. This process should also recognize and value the knowledge and practices of Andean mothers and their role as primary caretakers.

## Additional file

Additional file 1: List of questions for semi-structured interviews with mothers. (DOCX 14 kb)

## Abbreviations

WHO: World Health Organization

## Footnotes

1 Substances used to increase the milk supply.

2 Culturally defined illness. Symptoms include intense side and stomach pain and cough. Classified as hot and cured by cold.
