# Supplementary material for: Comparing Indigenous and public health infant feeding recommendations in Peru: opportunities for optimizing intercultural health policies
Source: J Ethnobiol Ethnomed. 2018 Nov 20;14:69. doi: 10.1186/s13002-018-0271-2 (PMC6245876; doi:10.1186/s13002-018-0271-2)
Supplement: Supplementary file 1 — List of questions for semi-structured interviews with mothers. (DOCX 14 kb) [file 13002_2018_271_MOESM1_ESM.docx]

List of questions for semi-structured interviews with mothers:

1. How old are you?
2. Where were you born?

(If in community) Did you always live here?

(If born somewhere else) How long did you live there and when did you move to community?

1. What should you eat while breastfeeding?
2. Should you eat more, less or the same while breastfeeding?
3. Are there foods, drinks or other things that are especially helpful in producing breast milk? (Ask what else until she says nothing else)

(If she mentions *hak’achu*) Who did you learn from and how did you obtain the *haka’chu*?

(if she does not mention the *hak’achu*) Have you heard about the *hak’achu* for producing more milk?

1. Are there foods, *mates* (infusions) or other things that you should not consume while you are breastfeeding? (ask what else until she says nothing else)
2. Should you drink water while you are breastfeeding? Why or why not?
3. Are there foods, drinks or other things that dry-up breast milk? (Ask what else until she says nothing else)
4. Where does breast milk come from? How is it formed in the body?
5. When should you start breastfeeding? (Prompt… immediately after birth, some hours after, a few days after. Why should you start then?
6. Should one feed the baby colostrum? Why or why not?
7. When should you start feeding the baby foods or drinks? Why at that time?
8. What foods or drinks should you feed to babies first? (ask what else until she says nothing else)
9. How long should you breastfeed? When should you stop breastfeeding? Why?
10. How do you stop breastfeeding? Is it easy, difficult?
11. What can you do so that the milk dries-up when you stop breastfeeding?
12. If you are sick can you pass on the illness to your infant through breast milk?
13. If the mother is sad or mad should she continue to breastfeed? Why or why not?
14. What illnesses affect babies? (ask what else until she says nothing else)

What causes x illness? (ask about each illness mentioned)

How do you know whether a baby has X illness? (Ask about each illness mentioned)

How do you cure x illness? (Ask about each illness mentioned)

1. How many children do you have?
2. How many boys? How many girls?
3. How many live now?

(If some died) Why did he/she die?

1. Where were your children born? (Prompt at home, at the health post, at the health center in Pisac, hospital in Cusco, somewhere else?
2. Who assisted you during birth? How did they assist you?
3. How long did you rest after birth?
4. What did you eat after birth?
5. Were your children born big, medium, or small size?
6. What was your diet like when you were pregnant?

(If she ate little) Were your children small?

(if she ate a lot) Were your children big?

Does the amount of food that you eat while you are pregnant matter?

1. What do you think about the services at the health post and health center?
2. Are mothers now a days raising their children in the same way as mothers before, your mother or grandmother? Why or why not?
